# Pregnancy and Peripartum Multidisciplinary Management in Wolfram Syndrome Type 1: A Case Report

**DOI:** 10.3390/diagnostics16081117

**Published:** 2026-04-08

**Authors:** Gema Esteban-Bueno, María Luz Serrano Rodríguez

**Affiliations:** 1 Clinical Management Unit Almería Periferia, Almería Health District, Andalusian Health Service (SAS), 04009 Almeria, Spain; 2 Spanish Association for Research and Support to Wolfram Syndrome, 04120 Almeria, Spain; 3 Hospital Universitario Fundación Alcorcón, 28922 Alcorcon, Spain; marialuz.serrano@salud.madrid.org

**Keywords:** Wolfram syndrome type 1, pregnancy, peripartum management, external cephalic version, cesarean delivery, combined spinal–epidural anesthesia, neuraxial anesthesia, diabetes insipidus, neurogenic bladder, case report

## Abstract

**Background/Objectives:** Wolfram syndrome type 1 (WS1) is a rare, progressive, multisystem neurodegenerative disorder characterized by diabetes mellitus, optic atrophy, diabetes insipidus, and sensorineural hearing loss. As survival has improved, an increasing number of affected women are reaching reproductive age. However, evidence on pregnancy and peripartum management in WS1 remains scarce, and practical guidance is limited. This case report describes the multidisciplinary management of pregnancy and delivery in a woman with genetically confirmed WS1 and highlights key considerations for peripartum care. **Case Presentation:** A woman with genetically confirmed WS1 and long-standing multisystem involvement, including diabetes mellitus, diabetes insipidus, neurogenic bladder requiring frequent self-catheterization, progressive neurologic manifestations, and severe sensory impairment, achieved pregnancy through assisted reproduction with oocyte donation and was closely monitored by a multidisciplinary team. Due to persistent breech presentation, a planned external cephalic version was performed at 37 + 5 weeks’ gestation with immediate availability for cesarean delivery. After unsuccessful attempts, cesarean delivery was performed under combined spinal–epidural anesthesia. Peripartum management focused on strict glycemic control, careful monitoring of fluid balance and urine output, neuraxial anesthesia with proactive hemodynamic management, precautions related to the cochlear implant, and tailored communication strategies. Postpartum recovery was favorable, although anemia on postoperative day 1 required transfusion of one unit of packed red blood cells and intravenous iron therapy. **Discussion and Conclusions:** Pregnancy in WS1 represents a high-risk clinical scenario because of the coexistence of endocrine, urologic, and neurologic comorbidities, while published evidence on peripartum management remains limited. This case supports an individualized, multidisciplinary approach to obstetric and anesthetic planning and the use of a practical framework to optimize peripartum management and enhance maternal–fetal safety in this rare condition.

## 1. Introduction

Wolfram syndrome type 1 (WS1), historically referred to as DIDMOAD, is a rare, progressive, autosomal recessive multisystem disorder characterized primarily by juvenile-onset diabetes mellitus and optic atrophy, often associated with central diabetes insipidus and sensorineural hearing loss [1,2]. The disease usually begins in childhood, with diabetes mellitus often representing the earliest manifestation, followed by optic atrophy in the first decade of life, although the clinical course is heterogeneous and additional manifestations may develop progressively over time [1,2,3,4,5]. WS1 is clinically suspected on the basis of a suggestive phenotype and confirmed by molecular genetic testing. There is currently no curative treatment; therefore, management is based on long-term multidisciplinary care focused on metabolic control, hormone replacement when indicated, sensory rehabilitation, surveillance of urologic complications, and monitoring of neurologic progression [1,2,6].

Most cases of WS1 are associated with biallelic pathogenic variants in *WFS1*, located on chromosome 4p16.1, which encodes wolframin, a transmembrane glycoprotein primarily localized to the endoplasmic reticulum [1,3,4,5]. Wolframin is involved in endoplasmic reticulum homeostasis, calcium handling, protein processing, and cellular responses to endoplasmic reticulum stress. Loss of wolframin function increases the vulnerability of pancreatic beta cells, retinal ganglion cells, cochlear cells, and specific neuronal populations, thereby contributing to the multisystem phenotype of WS1 [1,3,4]. In recent years, the concept of the “*WFS1* spectrum” has been refined, encompassing overlapping phenotypes, including Wolfram-like forms, and highlighting substantial variability in age at onset, disease progression, and neurologic involvement [3,4,5]. Although genotype–phenotype correlations do not allow deterministic prediction at the individual level, molecular characterization and variant-type analysis provide a useful framework for contextualizing disease severity and progression and supporting longitudinal follow-up [3,4,5]. Recent consensus statements also emphasize the need for genetic confirmation, early multidisciplinary assessment, and individualized management plans designed to anticipate disease-related complications [6].

Beyond the DIDMOAD core, the clinical spectrum frequently includes urologic involvement, such as neurogenic bladder, urinary retention or incontinence, recurrent urinary tract infections, and potential upper urinary tract deterioration, as well as neuropsychiatric manifestations and progressive neurologic complications, including ataxia, neuropathy, and possible autonomic dysfunction, all of which have a significant impact on quality of life and prognosis [1,6,7,8]. In practice, this combination requires coordinated follow-up across endocrine, urologic, and neurologic domains, with disease progression taken into account to guide therapeutic decisions over time [1,6,7].

As survival improves and more patients reach reproductive age, pregnancy in WS1 has become increasingly relevant in clinical practice, although it remains uncommon and is generally considered high risk [1,2,9,10]. This classification is based on several factors: (i) insulin-dependent diabetes mellitus requiring tight metabolic control; (ii) diabetes insipidus with potential fluid, electrolyte, and volume instability; (iii) urologic dysfunction with infectious implications and perioperative management challenges; and (iv) possible neurologic and/or autonomic involvement, which may influence hemodynamic tolerance and stress responses [1,2,9,10]. In addition, gonadal dysfunction and/or infertility may coexist and add complexity to the reproductive pathway, prompting the use of assisted reproductive technologies in some patients [1,11].

General case-based reviews have also summarized the broader clinical manifestations, genetics, pathophysiology, and potential therapies of WS1 [12]. However, available evidence on pregnancy and delivery in WS1 is largely based on case reports and small reviews, with heterogeneity in the level of peripartum detail and limited standardization of clinical reasoning and decision-making [9,10]. Although reviews and recommendations focused on diagnosis and longitudinal follow-up of the syndrome exist, operational gaps persist regarding pregnancy surveillance, planning of the mode of delivery (vaginal vs. cesarean delivery), and obstetric analgesia/anesthesia in the context of multisystem involvement and potential neurologic progression [1,2,6]. Consequently, there remains a practical need for reproducible peripartum management strategies that integrate individualized endocrine, neurologic, and urologic risks.

In this context, this case report presents the multidisciplinary management of pregnancy and delivery in a woman with WS1 and molecular confirmation of a *WFS1* variant, long-standing multisystem involvement, and severe sensory disability. The aim is to provide a practical, clinically applicable framework to enhance maternal–fetal safety in WS1, including key considerations and a potential checklist/algorithm for peripartum planning, and to clarify what this case adds to the available literature [6,9,10].

## 2. Case Presentation

### 2.1. Patient Information and Medical History

A 31-year-old woman with Wolfram syndrome type 1 (WS1; DIDMOAD), diagnosed in childhood, had been under specialized follow-up since the age of 15 years. There was no known family history of WS1; the patient was an only child, and no affected relatives were reported. The diagnosis of WS1 was established in 2004 and was subsequently genetically confirmed, as documented in the genetics report. Non-autoimmune insulin-dependent diabetes mellitus was diagnosed at 4 years of age. A history of symptomatic hypoglycemia was reported, including a severe episode approximately 15 years earlier that involved loss of consciousness and required urgent medical attention. During pregnancy, intermittent clinically significant hypoglycemic episodes occurred, with autonomic and neuroglycopenic symptoms including sweating, paresthesias, numbness of the hands and tongue, and drowsiness; some episodes required third-party assistance. The patient was therefore advised not to remain alone because of the risk of syncope.

Reduced visual acuity also began at 4 years of age, and optic atrophy was diagnosed at 6 years of age. No associated retinal dystrophy was identified, and ophthalmologic follow-up showed no diabetic retinopathy. Central diabetes insipidus developed at 9 years of age. Hearing loss began at 13 years of age, with sensorineural deafness diagnosed at 15 years of age. Hearing aids were initially used, and cochlear implantation was performed at 24 years of age. The patient reported suboptimal performance of the cochlear implant due to a defect in the internal component; therefore, the progression of hearing impairment could not be reliably interpreted as reflecting sensorineural deterioration alone. In the hospital setting, communication usually required support from relatives or an interpreter, and staff needed to speak at close range.

There was no obesity and no known cardiac or hepatic abnormality. Urologic involvement began at 18 years of age with a neurogenic bladder requiring very frequent intermittent self-catheterization (approximately 12 times per 24 h, although this varied according to urine output and diabetes insipidus control). Since initiation of self-catheterization, no recurrent urinary tract infections had occurred. One episode of severe urinary tract infection in 2018 was documented in the context of advice to increase the frequency of bladder emptying because of diabetes insipidus and was not attributed to self-catheterization itself.

Regarding neurologic involvement, gait instability was reported from 24 years of age. A chronic irritative cough had been present since 15–16 years of age, initially interpreted as “dry pharyngitis”, with throat itching. Over the previous two years, occasional choking episodes with liquids or solids, especially dry foods (e.g., toasted bread or nuts), were reported, occurring approximately once per month without aspiration pneumonia. At 27 years of age, ataxia and dysmetria were documented; from the same age, sialorrhea and hyporeflexia also developed, all in the setting of severe sensory disability. No dysarthria or anosmia was reported. An absent gag reflex had been documented since 2018. Orthostatic-type dizziness was reported since 2023 (or possibly slightly earlier), particularly when standing up quickly.

A history of hypogonadism and infertility was documented, and hormonal contraception had been used since the age of 16 years. Attempts to conceive began at 29 years of age. In 2023, following the patient’s wish to conceive, which had initially been deferred because of participation in a clinical trial, evaluation was performed at an assisted reproduction unit. Ovarian insufficiency was identified, with an almost undetectable anti-Müllerian hormone level (AMH 0.01) and no functional ovarian reserve on antral follicle count; therefore, in vitro fertilization with oocyte donation was indicated. The partner’s karyotype was normal. Accordingly, the main indication for oocyte donation was absent ovarian reserve. Surgical history included adenoidectomy/tonsillectomy in childhood, surgery for orthopedic trauma, and cochlear implantation, all under general anesthesia, with no reported anesthetic or surgical complications. A perianal procedure had also been performed under neuraxial anesthesia two years earlier without incident. At the time of pregnancy, long-term treatment included insulin degludec (long-acting) and desmopressin.

### 2.2. Genetic Testing

Genetic confirmation was obtained from the patient’s clinical genetics report. Molecular analysis of *WFS1* identified a homozygous pathogenic nonsense variant, NM_006005.3:c.1558C>T (p.Gln520Ter), located in exon 8. This variant introduces a premature termination codon and is predicted to result in loss of function, with marked reduction or absence of functional wolframin, which is a recognized disease mechanism in Wolfram syndrome type 1.

The variant had been identified previously as part of routine clinical diagnostic testing. Although the full laboratory workflow was not available to the authors because the analysis had been performed in the clinical setting before preparation of this report, the result was issued within a standard diagnostic setting and interpreted in light of the patient’s phenotype and the established disease association of *WFS1* loss-of-function variants.

Variant nomenclature was revised according to HGVS recommendations using the reference transcript NM_006005.3. Throughout the manuscript, the human gene symbol *WFS1* is presented in italics in accordance with standard nomenclature conventions.

### 2.3. Reproductive Planning and Pregnancy Course

In the context of WS1 (autosomal recessive inheritance) and following evaluation at an assisted reproduction unit for infertility secondary to ovarian insufficiency with absent functional ovarian reserve, in vitro fertilization with oocyte donation was chosen. Pregnancy was achieved through this approach and was monitored from early gestation by a multidisciplinary team (Obstetrics, Endocrinology, Anesthesiology, Ophthalmology, Neurology, and Urology).

Endocrinologic evaluation documented stable pregestational diabetes control and no known diabetic microvascular complications. Central diabetes insipidus, diagnosed in childhood, had not caused relevant decompensations or prior hospital admissions; therefore, close monitoring of both diabetes mellitus and diabetes insipidus during pregnancy, the peripartum period, and the puerperium was considered a priority. Autoimmune thyroiditis with preserved thyroid function was recorded. During pregnancy, intermittent episodes of symptomatic hypoglycemia were reported, some requiring external assistance, which further supported the need for close surveillance and dynamic treatment adjustments.

Urologic evaluation documented the need for very frequent intermittent self-catheterization (≈12 times/24 h; estimated and variable in relation to diabetes insipidus and urine output). No urinary tract infections had occurred since initiation of self-catheterization; one episode of severe urinary tract infection in 2018 was reported in association with recommendations for more frequent bladder emptying in the context of diabetes insipidus. After multidisciplinary discussion, the urologic status was not considered an indication for early delivery, and the timing of birth was determined by obstetric criteria.

Neurologic assessment shortly before delivery showed preserved language and no relevant cranial nerve abnormalities except for severe visual impairment (light perception only). No focal motor deficits were observed. A mild distal reduction in vibratory sensation in the lower limbs was described; coordination was considered preserved, and gait showed no striking abnormalities on examination, which was overall interpreted as clinical stability relative to the patient’s baseline status. A prior electromyography study had shown mild chronic neurogenic changes bilaterally in S2–S4 territories, compatible with early peripheral neuropathy in the context of WS1. Orthostatic-type dizziness and episodic tension-type headache were reported. From a neurologic perspective, no contraindications to attempting vaginal delivery were identified, although pelvic floor weakness was mentioned as a possible factor contributing to reduced expulsive effectiveness.

Ophthalmologic evaluation confirmed bilateral optic atrophy with no evidence of diabetic retinopathy at the time of evaluation.

Overall, the obstetric course was favorable, with ultrasound follow-up showing fetal growth appropriate for gestational age. In the third trimester, breech presentation was identified; therefore, multidisciplinary meetings were held to agree on the delivery plan. A planned external cephalic version (ECV) at 37 + 5 weeks’ gestation was considered appropriate because ECV is generally performed after 36–37 weeks [13], when spontaneous version is less likely, while still allowing the procedure to be undertaken before the onset of labor. In this case, the chosen timing also allowed the maneuver to be scheduled in a controlled multidisciplinary setting with immediate access to cesarean delivery if needed [14]. It was further supported by concerns about continued fetal growth in the setting of maternal diabetes and by the risk of urinary complications related to frequent self-catheterization due to neurogenic bladder.

### 2.4. Preanesthetic Assessment and Peripartum Planning

At the preanesthetic assessment, the patient’s weight was 67 kg and her height was 1.63 m. Airway evaluation showed Mallampati class II, mouth opening >3 cm, and mild retrognathia. These findings, together with pregnancy-related physiological changes, were considered factors that could increase the complexity of airway management if general anesthesia became necessary.

No contraindications to epidural labor analgesia were identified, and neuraxial anesthesia was prioritized as the technique of choice in the event of cesarean delivery, as it was considered safer than general anesthesia in this patient. Nevertheless, an alternative plan was established in the event that conversion to general anesthesia was required, including preparation of difficult-airway equipment and a rescue strategy.

Given possible neuromuscular/neurologic involvement in the context of a multisystem neurodegenerative disorder, it was planned to minimize the use of neuromuscular blocking agents whenever possible. If required, neuromuscular monitoring and careful dose titration were considered essential due to the risk of unpredictable response and prolonged blockade. Succinylcholine was also considered undesirable because of the potential risk of hyperkalemia in certain contexts of denervation or muscle weakness.

Across the peripartum period, placement of a urinary catheter with strict urine output monitoring was emphasized, together with tight glycemic control coordinated with Endocrinology using a peripartum protocol. The patient used continuous glucose monitoring (CGM), which facilitated metabolic follow-up during pregnancy and the postpartum period. Minimization of preoperative fasting was also recommended given the coexistence of diabetes mellitus and diabetes insipidus.

Because the patient had a cochlear implant, preferential use of bipolar electrocautery was planned [15]. No device-related incidents or interference occurred during the procedure.

### 2.5. Delivery/Cesarean Section and Anesthetic Management: Intraoperative Course

The patient was admitted electively for external cephalic version (ECV), with the option of immediate cesarean delivery if the maneuver failed. In the obstetric unit, maternal–fetal monitoring was performed, two peripheral intravenous lines were placed, and the intrapartum glycemic control protocol prescribed by Endocrinology was implemented. A urinary catheter was inserted with hourly urine output monitoring, and blood type and antibody screening were requested. Mechanical thromboprophylaxis was initiated using intermittent pneumatic compression and/or elastic stockings [16].

In the operating room, standard monitoring was applied (ECG, pulse oximetry, noninvasive blood pressure, and temperature). Oxygen was administered via nasal cannula with continuous capnography monitoring. To reduce the risk of neuraxial anesthesia-associated hypotension, left uterine displacement (≈15°), crystalloid co-loading, vasopressor administration according to hemodynamic status (total ephedrine dose, 9 mg), and careful titration of the local anesthetic were used [17].

A combined spinal–epidural (CSE) technique was performed to optimize anesthetic control and to provide an epidural catheter for tailored titration in the event that cesarean delivery was required. The procedure was performed in the left lateral decubitus position, thereby minimizing the risk of orthostatic-type dizziness reported by the patient. The epidural space was identified at L3–L4 and, using a combined spinal–epidural set, intrathecal puncture was performed with a 27-gauge pencil-point needle; hyperbaric bupivacaine 5 mg (0.5%) with fentanyl 10 µg was administered. The epidural catheter was then placed. The technique was uneventful, with good cooperation, hemodynamic stability, and a satisfactory sensory block.

Ultrasound-guided ECV was attempted; after three unsuccessful attempts, cesarean delivery was indicated due to persistent breech presentation and was performed immediately, in accordance with the previously agreed plan and after confirming patient understanding with the support of her companion.

Antibiotic prophylaxis with cefazolin 2 g IV was administered. After confirming an adequate sensory block, the sterile surgical field was prepared and cesarean delivery was performed. A male neonate weighing 3490 g was delivered, with Apgar scores of 9/9. Initial neonatal assessment was normal. Manual placental extraction was performed. After cord clamping, uterotonic agents (carbetocin 100 µg IV and methylergonovine 0.2 mg IM) were administered, achieving adequate uterine tone.

For intraoperative analgesia and adjuvant medication, dexketoprofen 50 mg IV and ondansetron 4 mg IV were administered. Through the epidural catheter, levobupivacaine 0.5% (2 mL + 2 mL) was administered to maintain an adequate sensory level (T7–T6).

During the procedure, the patient experienced an acute anxiety episode that resolved with propofol 10 mg IV, maintaining spontaneous ventilation without need for additional respiratory support. The partner remained present throughout the procedure to facilitate communication and support, and skin-to-skin contact was encouraged after birth.

Estimated blood loss was 1000 mL. Urine remained clear, and hourly urine output remained adequate.

### 2.6. Postpartum and Follow-Up

Immediate postoperative recovery took place in the obstetric recovery area under monitoring and postanesthetic surveillance, with the patient’s partner continuously present and skin-to-skin contact with the newborn maintained. After 3 h, the patient was transferred to the ward, with hemodynamic and respiratory stability, appropriate uterine involution, satisfactory pain control, and motor recovery according to the Bromage scale.

On postoperative day 1, laboratory testing showed anemia requiring transfusion of one unit of packed red blood cells, followed by intravenous iron administration (ferric carboxymaltose). Breastfeeding was maintained with occasional formula supplementation. Thromboprophylaxis with enoxaparin 40 mg was administered according to protocol. Endocrinology continued close glucose monitoring and dose adjustment during the puerperium.

The neonate was a term male infant born at 37 + 5 weeks’ gestation, with a birth weight of 3490 g, length of 50 cm, and head circumference of 35 cm. He was admitted to the neonatal unit at 24 h of life because of feeding difficulties and suspected sepsis. Initial lethargy and mild hypotonia improved over the following hours, with recovery of normal tone and adequate oral feeding by 36 h of life. Mild jaundice remained below the phototherapy threshold, pulmonary ultrasound was normal, and transient hypocalcemia was documented. He passed pulse oximetry screening for critical congenital heart disease and bilateral hearing screening and was discharged back to the maternity ward after clinical improvement.

According to postpartum clinical follow-up, the patient remained neurologically stable after pregnancy, with no new deficits or worsening of the neurologic manifestations already present during gestation.

### 2.7. Case Timeline

**Figure 1:**
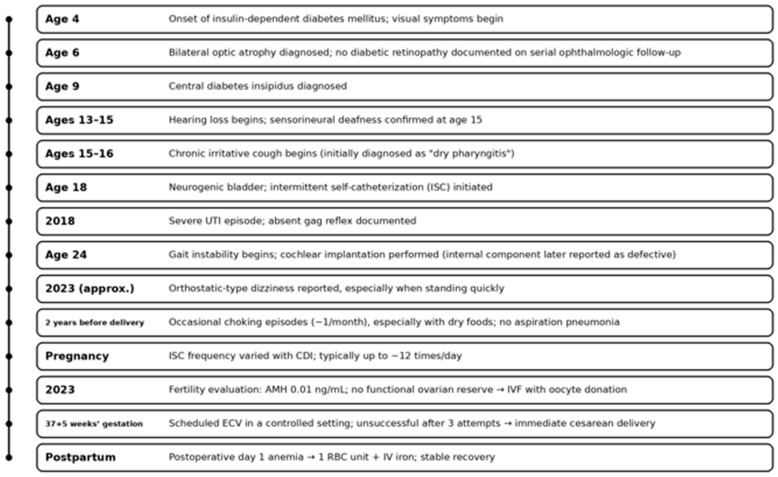
Clinical timeline of the patient with Wolfram syndrome type 1 (WS1). Key clinical milestones from childhood to the postpartum period, including endocrine, sensory, urologic, and neurologic manifestations, as well as major obstetric events. Abbreviations: AMH, anti-Müllerian hormone; CDI, central diabetes insipidus; ECV, external cephalic version; ISC, intermittent self-catheterization; IVF, in vitro fertilization; IV, intravenous; RBC, red blood cells; UTI, urinary tract infection.

The key clinical milestones from childhood to the postpartum period are summarized in Figure 1.

## 3. Discussion

Wolfram syndrome type 1 (WS1) is a rare, progressive, multisystem disorder, and improved survival has allowed an increasing number of patients to reach reproductive age [1,2]. However, evidence on pregnancy and delivery management in WS1 remains limited and is largely based on case reports and small series, with little standardization of peripartum and anesthetic planning [9,10]. In this context, the present case provides a detailed description of the clinical reasoning and multidisciplinary management focused on maternal–fetal safety, together with a practical framework to support peripartum planning (Table 1 and Table 2).

Pregnancy in WS1 can be considered a major physiologic stressor due to the coexistence of endocrine, urologic, and neurologic comorbidities [1,2,4]. The combination of insulin-dependent diabetes mellitus and central diabetes insipidus entails an increased risk of metabolic decompensation, fluid and electrolyte disturbances, and hemodynamic variability, potentially accentuated during labor and the perioperative period [1,2,9,18]. With respect to pregestational diabetes, current standards recommend structured peripartum protocols (insulin/dextrose regimens, minimization of fasting, intensive monitoring, and early postpartum dose adjustment) [19,20,21]. Although no WS1-specific guidance exists for central diabetes insipidus in pregnancy, endocrine guidance for inpatient management of cranial diabetes insipidus (strict fluid balance recording, close urine output monitoring, and prevention of dysnatremias through individualized desmopressin adjustment) provides a directly applicable framework for the peripartum setting [18]. In addition, urologic involvement is common and may present as neurogenic bladder requiring self-catheterization, with an increased risk of recurrent urinary tract infections, adding complexity to urine output assessment and infectious complication prevention [7,8]. This is compounded by possible progressive neurologic involvement, described in advanced stages of the syndrome, which may include autonomic dysfunction and alter physiologic stress responses [2,4,6].

From a genetic standpoint, marked phenotypic heterogeneity has been described in WS1 and the broader *WFS1*-associated spectrum, partly related to the functional impact of variants and the degree of wolframin loss [1,3,5]. This framework, together with clinical assessment, may help contextualize multisystem evolution and anticipate potential risks in high-stress situations such as pregnancy and delivery [3,4]. In this regard, recent analyses addressing phenotypic correlations of neurologic manifestations may help structure follow-up and clinical stratification in WS1, particularly when individualized peripartum planning is required [22].

In the present case, obstetric planning included a scheduled external cephalic version (ECV) at 37 + 5 weeks’ gestation, with immediate cesarean availability if the maneuver failed. Although WS1-specific literature on ECV is virtually nonexistent, indications and safety standards for term ECV are well established in general obstetric guidelines [13]. Moreover, anesthesiology recommendations on anesthetic support during ECV (appropriate setting selection, preparedness, and management of adverse events) support scheduling the procedure in a controlled environment with the capacity for immediate conversion to cesarean delivery when the overall maternal–fetal risk profile favors minimizing uncertainty [14].

Early anesthetic assessment was key to identifying specific risks and guiding the peripartum strategy. Predictors of potentially difficult airway reinforced the preference for neuraxial techniques, minimizing the likelihood of general anesthesia. In the absence of WS1-specific recommendations, management should be individualized according to multisystem involvement and aligned with general obstetric anesthesia principles (anticipatory planning, neuraxial techniques when appropriate, and preparation for difficult airway management) [23].

The combined spinal–epidural (CSE) technique offered advantages in this setting by allowing low intrathecal dosing with subsequent epidural titration, thereby adapting to clinical and surgical evolution. Intraoperative management incorporated preventive measures targeting key risks, including active prophylaxis of neuraxial anesthesia-associated hypotension and protocolized vasopressor use, consistent with international consensus for cesarean delivery under spinal anesthesia [17]; respiratory monitoring with capnography during oxygen therapy; and strict urine output monitoring via bladder catheterization, particularly relevant in the setting of diabetes insipidus and neurogenic bladder [18].

A distinctive aspect of this case was severe sensory disability and the presence of a cochlear implant, which influenced both communication and specific technical precautions in the operating room. Adapted communication, with continuous support from the partner, was essential to reduce anxiety and facilitate cooperation. Regarding the cochlear implant, available literature supports heightened caution with electrosurgery and, when possible, preference for techniques that minimize interference and device-related risk [15]. The intraoperative anxiety episode, resolved with a low dose of IV propofol without respiratory compromise, underscores the importance of anticipating and proactively addressing psychological and communication needs—an aspect seldom discussed in published reports of pregnancy and delivery in WS1 [9,10].

The postpartum period constitutes another critical phase in patients with WS1. In this case, postoperative day 1 anemia requiring transfusion and intravenous iron therapy highlights the need for close surveillance even after an initially stable intraoperative course. Continuation of thromboprophylaxis and metabolic control during the puerperium was consistent with general obstetric guidance for venous thromboembolism prevention during pregnancy and the postpartum period [16,24].

**Figure 2:**
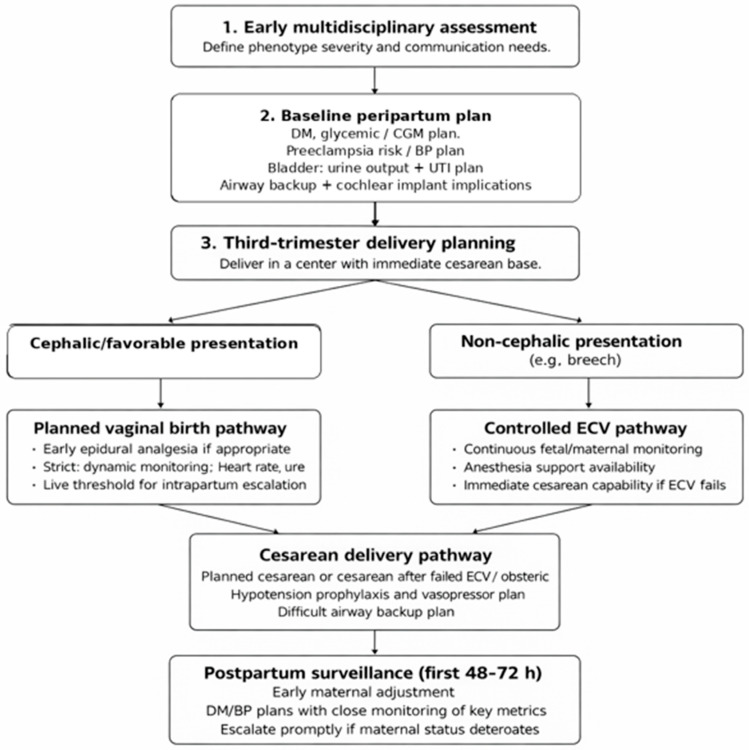
Proposed peripartum planning algorithm for pregnancy complicated by Wolfram syndrome type 1 (WS1): a multidisciplinary, risk-based approach. Abbreviations: BP, blood pressure; CGM, continuous glucose monitoring; DM, diabetes mellitus; ECV, external cephalic version; UTI, urinary tract infection.

Overall, this case reinforces the need for a multidisciplinary, individualized approach to pregnancy in WS1, integrating coordinated care by Obstetrics, Anesthesiology, and Endocrinology, and involving Urology and Neurology according to phenotype. To enhance clinical applicability, key considerations are synthesized in a practical checklist (Table 1) and a domain-based peripartum plan (Table 2). A proposed peripartum planning algorithm is presented in Figure 2. To support real-time decision-making, operational alert and escalation criteria (“red flags”) for the intrapartum period and immediate puerperium are also provided in Table S1. This report is limited by its single-case design; therefore, generalizable conclusions cannot be drawn. Nonetheless, given the rarity of the syndrome and the lack of condition-specific guidelines, a detailed and structured description of management may be useful for other teams facing similar situations [9,10].

## 4. Conclusions

Pregnancy in Wolfram syndrome type 1 should be considered high risk due to the combination of endocrine comorbidities (diabetes mellitus and diabetes insipidus), urologic involvement, and neurologic disease, with potential for fluid and electrolyte disturbances, hemodynamic variability, and infectious complications during the peripartum period. In this case, a structured, reproducible plan based on multidisciplinary coordination, a peripartum glycemic control protocol, strict fluid balance and urine output monitoring, and a neuraxial-first anesthetic strategy (with active hypotension prophylaxis and a difficult-airway rescue plan) enabled safe peripartum management, including scheduled ECV in a setting with immediate cesarean capability. In addition, severe sensory disability and the presence of a cochlear implant require systematic communication adaptations and electrosurgery precautions, often underappreciated factors that may be decisive for perioperative safety and patient comfort in WS1.

## Figures and Tables

**Table 1:** Practical checklist for peripartum planning and delivery in Wolfram syndrome type 1 (WS1).

Area	Items to Assess	Practical Considerations
Endocrine assessment	Diabetes mellitus and central diabetes insipidus (CDI)	Protocolized intrapartum and postpartum glycemic control coordinated with Endocrinology; minimize preoperative fasting; adjust fluids and desmopressin according to fluid balance.
Fluid balance	Risk of electrolyte disturbances	Urinary catheterization with hourly urine output; close monitoring of hydration status and urine volume.
Urologic involvement	Neurogenic bladder, intermittent self-catheterization, recurrent UTI	UTI prevention; strict urine output monitoring; coordinate with Urology when there is relevant history or when complications arise.
Neurologic/autonomic assessment	Progressive neurologic involvement, possible dysautonomia	Assess orthostatic symptoms and hemodynamic tolerance; anticipate possible variability in the stress response during labor and surgery.
Anesthesia plan	Airway and anesthetic technique	Prefer titratable neuraxial techniques; identify potential difficult airway early; establish a backup plan if general anesthesia is required.
Intraoperative monitoring	Hemodynamic and respiratory stability	Actively prevent neuraxial hypotension; monitor respiration during oxygen administration; maintain continuous vital sign surveillance.
Implantable devices	Cochlear implant or other devices	Prefer bipolar electrosurgery; if monopolar electrosurgery is required, follow manufacturer and institutional precautions (place the return pad away from the implant and minimize current flow near the head and neck).
Communication and support	Severe sensory impairment (deafblindness)	Use an adapted communication plan; provide anticipatory guidance; facilitate partner presence to support communication and reduce anxiety.
Postpartum	Thromboembolic and metabolic risk	Risk-based VTE prophylaxis; continued glucose and fluid balance monitoring during the puerperium; multidisciplinary follow-up.

Abbreviations: WS1, Wolfram syndrome type 1; CDI, central diabetes insipidus; UTI, urinary tract infection; VTE, venous thromboembolism.

**Table 2:** Multidisciplinary peripartum management plan by clinical domain in Wolfram syndrome type 1 (WS1).

Domain	Key Risk/Issue in WS1	Minimum Monitoring Set	Recommended Actions	Lead Team(s) and Timing
Obstetrics/Delivery planning	Breech presentation; need for controlled decision-making; limited WS1 pregnancy evidence.	Maternal–fetal monitoring; ultrasound confirmation of presentation.	Plan ECV in a setting with immediate access to cesarean delivery; pre-briefed contingency plan; shared decision-making.	Obstetrics, Anesthesiology Late third trimester; day of ECV/delivery.
Pregestational diabetes mellitus	Glycemic instability during fasting/labor/surgery; postpartum insulin requirement changes.	CGM trends + capillary confirmation per protocol; ketones if clinically indicated.	Standardized intrapartum/perioperative insulin–dextrose protocol; avoid prolonged fasting; prompt treatment of hypo-/hyperglycemia; early postpartum dose adjustment.	Endocrinology, Anesthesiology, Obstetrics, Nursing Admission; intrapartum/intraop; first 24–72 h postpartum.
Central diabetes insipidus (CDI)	Dysnatremia risk; dehydration or water intoxication; high urine output confounds fluid balance.	Strict input/output; hourly urine output; clinical volume status; serum sodium/osmolality per protocol.	Individualized desmopressin plan; avoid unnecessary free-water overload; maintain euvolemia; proactive correction strategy for hypo-/hypernatremia; minimize fasting.	Endocrinology, Anesthesiology, Nursing Admission; periop; early postpartum.
Urology (neurogenic bladder)	Retention/high residuals; history of severe UTI (2018); complicates CDI/fluid management.	Hourly urine output; urine appearance; temperature/symptoms; urinalysis/culture if symptomatic.	Foley catheter perioperatively with strict output recording; aseptic technique; plan for catheter removal and/or resumption of intermittent self-catheterization (frequency variable in pregnancy, typically up to ~12/day); evaluate for UTI if symptomatic.	Obstetrics, Nursing; Urology as needed Admission; intraop; postpartum.
Neurology/autonomic features	Orthostatic symptoms; possible autonomic dysfunction; variable hemodynamic response.	Blood pressure/heart rate trends; symptoms; vigilance after neuraxial anesthesia.	Left uterine displacement; avoid abrupt position changes; vasopressor readiness; individualized second-stage management if reduced expulsive effort.	Anesthesiology, Obstetrics; Neurology as needed Intrapartum/intraop; immediate postpartum.
Bulbar symptoms/aspiration risk	Choking history; weak cough; aspiration risk if general anesthesia required.	Respiratory rate, SpO_2_; capnography when oxygen is administered; airway reflex history.	Prefer neuraxial techniques; if general anesthesia is required, use aspiration precautions and fully awake extubation; postoperative respiratory observation.	Anesthesiology, Obstetrics, Nursing Preop planning; intraop; PACU.
Airway management	Potential difficult airway (pregnancy-related changes and craniofacial features); high risk if general anesthesia is required.	Standard airway assessment; equipment checks.	Difficult-airway cart and backup plan available; experienced staff present; avoid general anesthesia when feasible.	Anesthesiology Preop; intraop.
Neuromuscular blocking agents (if general anesthesia)	Unpredictable sensitivity/prolonged blockade possible in neurodegenerative context.	Neuromuscular monitoring (train-of-four) if paralytics are used.	Minimize/avoid neuromuscular blocking agents when possible; if required, titrate with train-of-four monitoring; consider avoiding succinylcholine; ensure full reversal and safe extubation criteria.	Anesthesiology Intraop.
Hemodynamic management (neuraxial anesthesia)	Neuraxial hypotension affecting uteroplacental perfusion.	Frequent noninvasive blood pressure measurements; symptoms; fetal monitoring.	Prophylaxis for neuraxial hypotension (left tilt, fluid co-load); vasopressor per protocol; titratable technique (e.g., CSE + epidural top-ups).	Anesthesiology, Obstetrics During neuraxial; intraop.
Cochlear implant/electrosurgery	Potential device interference/damage; communication limitations.	Device presence documented; OR checklist.	Prefer bipolar electrosurgery; if monopolar electrosurgery is required, follow manufacturer and institutional precautions.	Obstetrics (surgery), OR Nursing, Anesthesiology Intraop.
Communication and sensory impairment (deafblindness)	Miscommunication → anxiety and reduced cooperation; perioperative distress.	Confirm preferred communication method; reassess comfort/anxiety.	Pre-brief the patient and partner; allow partner presence when feasible; use agreed communication aids; plan proactive anxiolysis.	Obstetrics, Anesthesiology, Nursing Preop; intraop; postpartum.
Thromboprophylaxis	Increased VTE risk (cesarean + reduced mobility/complex comorbidity).	Clinical VTE risk assessment; bleeding monitoring.	Mechanical prophylaxis periop; pharmacologic prophylaxis postpartum per institutional protocol.	Obstetrics, Anesthesiology, Nursing Admission/intraop; postpartum.
Postpartum surveillance	Metabolic shifts; bleeding/anemia; urinary management; pain control.	Clinical bleeding/uterine tone; complete blood count, if indicated; glucose trends; urine output; pain scores.	Early detection and treatment of anemia; continue endocrine protocols; transition plan for bladder management; multimodal analgesia and early mobilization.	Obstetrics, Endocrinology, Nursing Recovery unit and ward; first 48–72 h.

Abbreviations: WS1, Wolfram syndrome type 1; CDI, central diabetes insipidus; CGM, continuous glucose monitoring; ECV, external cephalic version; CSE, combined spinal–epidural; UTI, urinary tract infection; SpO_2_, peripheral oxygen saturation; VTE, venous thromboembolism; PACU, postanesthesia care unit.

## Data Availability

The data presented in this study are contained within the article. Additional information may be available from the corresponding author upon reasonable request, subject to ethical and privacy restrictions.

## Supplementary Materials

The following supporting information can be downloaded at: https://www.mdpi.com/article/10.3390/diagnostics16081117/s1, Table S1: Red flags and escalation criteria for peripartum care in Wolfram syndrome type 1 (WS1).
