# Supplementary material for: Pregnancy and Peripartum Multidisciplinary Management in Wolfram Syndrome Type 1: A Case Report
Source: Diagnostics (Basel). 2026 Apr 8;16(8):1117. doi: 10.3390/diagnostics16081117 (PMC13114596; doi:10.3390/diagnostics16081117)
Supplement: Supplementary file 1 [file diagnostics-16-01117-s001.zip › diagnostics-4219962-supplementary.pdf]

Table S1. Red flags and escalation criteria for peripartum care in Wolfram syndrome type 1 (WS1).

Domain	Red flags (examples)	Immediate actions (per local pathway)	Escalation / who to involve	Key refs
Pregestational diabetes mellitus	• Recurrent hypoglycemia (<70 mg/dL; <3.9 mmol/L) or any severe episode requiring assistance<br>• Persistent hyperglycemia (>180 mg/dL; >10 mmol/L for >2 h)<br>• Positive ketones, vomiting, abdominal pain, Kussmaul breathing, or clinical concern for DKA	• Confirm CGM with capillary/venous glucose as per protocol<br>• Treat hypoglycemia promptly (oral/IV glucose) and reassess every 15 min until resolved<br>• Review insulin/dextrose regimen; consider ketone testing and acid–base evaluation if indicated<br>• Avoid prolonged fasting; ensure postpartum dose reassessment early	Endocrinology + Anesthesiology/Obstetrics; consider ICU/Medicine if DKA suspected or unstable	(20,22,23)
CDI / fluids & electrolytes	• Rapidly rising or falling Na; Na >145 mmol/L or symptomatic hypernatremia<br>• Na <130 mmol/L or symptomatic hyponatremia<br>• Sustained polyuria (e.g., >300 mL/h or >2 mL/kg/h) with thirst, hypotension, tachycardia, or signs of hypovolemia<br>• New confusion, seizures, or severe headache	• Strict intake/output; hourly urine output in acute phase<br>• Check serum Na/osmolality urgently and repeat per protocol<br>• Review desmopressin timing/dose and fluid strategy; avoid inappropriate free-water administration<br>• Correct dysnatremia according to endocrine/obstetric pathways	Endocrinology + Anesthesiology; ICU if seizures, severe symptoms, or unstable Na	(13)
Hemodynamic instability (labor/neuraxial)	• Post-neuraxial hypotension (SBP <90 mmHg or >20% fall from baseline) especially with symptoms or fetal concerns<br>• Refractory hypotension despite initial measures<br>• Syncope/near-	• Left uterine displacement; optimize positioning<br>• Fluid co-load and vasopressor treatment per protocol<br>• Evaluate for alternative causes (bleeding, high spinal block, sepsis)<br>• Continuous fetal	Anesthesiology + Obstetrics; ICU if shock or refractory instability	(16,24)

	syncope or suspected autonomic instability	monitoring when applicable		
Respiratory compromise / aspiration risk	• SpO_2 <94% despite supplemental oxygen<br>• Abnormal capnography or rising CO_2 when monitored<br>• Inability to clear secretions, repeated choking/aspiration signs<br>• Reduced consciousness after sedatives/analgesics	• Escalate oxygen delivery and airway support as needed; consider noninvasive support per pathway<br>• Minimize additional sedatives; suction/positioning<br>• If general anesthesia is required, use aspiration precautions and awake extubation when safe	Anesthesiology; ICU/Respiratory support team if persistent compromise	(24)
Airway (if GA required)	• Difficult mask ventilation or failed intubation<br>• Cannot intubate/cannot oxygenate scenario	• Activate institutional difficult-airway algorithm early<br>• Use video laryngoscopy/supraglottic devices; call for help<br>• Prepare front-of-neck access per institutional pathway	Senior anesthesiologist + ICU; ENT if required	(24)
Urology / UTI / sepsis	• Fever ≥38 °C or systemic symptoms<br>• Flank pain, rigors, or suspected pyelonephritis<br>• Obstructed catheter/no urine output; worsening dysuria/urinary symptoms	• Check catheter patency; ensure aseptic technique<br>• Urinalysis/culture if symptomatic; start antibiotics per obstetric/UTI pathway<br>• Maintain strict urine output monitoring	Obstetrics + Urology as needed; Infectious Diseases/ICU if sepsis suspected	(7,8)
Bleeding / anemia	• Postpartum hemorrhage per obstetric criteria<br>• Ongoing bleeding with tachycardia/hypotension<br>• Symptomatic anemia or clinically significant Hb drop per local thresholds	• Activate PPH protocol; uterotonics and surgical hemostasis as indicated<br>• Type and screen/crossmatch; transfuse per institutional thresholds<br>• IV iron therapy when appropriate	Obstetrics + Anesthesiology; blood bank; ICU if massive transfusion	(24)
VTE / thromboembolism	• Unilateral leg swelling/pain; sudden dyspnea; chest pain; hemoptysis<br>• Persistent unexplained	• Urgent assessment and diagnostic imaging per pregnancy pathway<br>• Ensure mechanical prophylaxis; initiate anticoagulation if	Obstetrics + Medicine/Hematology; ICU if suspected PE with instability	(18,21)

	tachycardia or hypoxemia<br>• High-risk features (reduced mobility, prior VTE) with new symptoms	clinically indicated per protocol		
Cochlear implant / electrosurgery	• Need for extensive monopolar electrosurgery<br>• Suspected device malfunction or unexpected loss of function<br>• Communication barrier impacting immediate safety	• Prefer bipolar electrosurgery when feasible<br>• If monopolar electrosurgery is required, follow manufacturer and institutional precautions (grounding pad away from implant; minimize current path near **the head and neck**)<br>• Document device status; ensure communication support	Operating room team + Anesthesiology; ENT if malfunction suspected	(17)
Communication and anxiety (deafblindness)	• Escalating anxiety/panic interfering with cooperation<br>• Inability to confirm understanding in urgent decisions	• Use pre-agreed communication method; involve partner/interpreter<br>• Provide anticipatory guidance and reassurance<br>• Consider low-dose anxiolysis with continuous respiratory monitoring when appropriate	Anesthesiology + Obstetrics; mental health support if needed	(9,10,12)

Note: Thresholds are intended to support situational awareness and escalation; clinical decisions should follow institutional obstetric/endocrine/anesthesia pathways and be individualized to maternal–fetal status.
Abbreviations: CDI, central diabetes insipidus; CGM, continuous glucose monitoring; CO_2, carbon dioxide; DKA, diabetic ketoacidosis; ENT, ear, nose, and throat; Hb, hemoglobin; ICU, intensive care unit; IV, intravenous; Na, serum sodium; PE, pulmonary embolism; PPH, postpartum hemorrhage; SBP, systolic blood pressure; SpO_2, peripheral oxygen saturation; UTI, urinary tract infection; VTE, venous thromboembolism.
